# Combined Treatment with Zinc Aspartate and Intravenous Immunoglobulins (IVIGs) Ameliorates Experimental Autoimmune Encephalomyelitis (EAE)

**DOI:** 10.1155/2018/5982169

**Published:** 2018-09-26

**Authors:** Diana Straubel, Anja Thielitz, Annegret Reinhold, Kurt Grüngreiff, Dirk Reinhold

**Affiliations:** ^1^Institute of Molecular and Clinical Immunology, Otto-von-Guericke-University Magdeburg, Magdeburg, Germany; ^2^University Clinic of Dermatology and Venereology, Otto-von-Guericke-University Magdeburg, Magdeburg, Germany; ^3^Clinics of Gastroenterology, City Hospital Magdeburg, Klinikum Magdeburg GmbH, Magdeburg, Germany

## Abstract

Intravenous immunoglobulins (IVIGs) are widely used in replacement therapy of primary and secondary immunodeficiency disorders and in approved autoimmune indications. In addition, IVIG application is used off-label for treatment of other autoimmune diseases, e.g., multiple sclerosis (MS), an inflammatory autoimmune disorder with a clear T cell-mediated immune pathogenesis. The trace element zinc is shown to play a regulatory role in the maintenance of immune functions. Changes of zinc homeostasis affect both the innate and the adaptive immune system. On one hand, therapeutic zinc supplementation can normalize impaired immune functions due to zinc deficiency. On the other hand, therapeutic zinc supplementation is under consideration as a possible option to treat T cell-mediated autoimmune diseases. The aim of the present study was to investigate the influence of IVIG (Octagam®), zinc aspartate (Unizink®), and the combined application of both preparations in the experimental autoimmune encephalomyelitis (EAE), the animal model of MS. Therapeutic intraperitoneal application of zinc aspartate significantly diminished clinical signs during the relapsing-remitting phase of EAE in SJL/J mice. In contrast, IVIG given in a therapeutic manner did not influence the course of EAE. Interestingly, the combined application of both, IVIG and zinc aspartate, significantly reduced the severity of the disease during the acute and the relapsing-remitting phase of the EAE. Our data suggest that the combination of IVIG and zinc aspartate may have beneficial effects in autoimmune diseases, like MS. Further studies should verify the benefit of a controlled immunosuppressive therapy with IVIG and zinc for such diseases.

## 1. Introduction

The chronic autoimmune disease multiple sclerosis (MS) is the most frequent demyelinating disease of the central nervous system (CNS) with a prevalence of 0.1% in Northern America and Europe. MS can affect all functional systems of the CNS, leading to symptoms like weakness of one or several limbs, optic neuritis, cerebellar or brainstem dysfunction, sensory deficits, and cognitive impairment [1, 2]. The experimental autoimmune encephalomyelitis (EAE) is the accepted animal model of MS. EAE is characterized as a T cell-mediated autoimmune disease, driven by CNS inflammation, demyelination, and neuronal loss [3, 4].

The trace element zinc is shown to be essential for a wide range of physiological processes, including cell and tissue differentiation, proliferation, and apoptosis. Zinc is involved in the regulation of numerous structural and catalytic functions, in protein-protein interactions, and in signal transduction of several cell types [5–8]. An impairment of zinc homeostasis by genetic defects and/or zinc deficiency affects both the components of the innate and the adaptive immune system, whereas therapeutic zinc supplementation normalizes the diminished immune functions due to zinc deficiency [9]. In contrast, high dosages of zinc suppress functions of immune cells, particularly of T cells [6, 8, 10, 11].

Based on these observations, therapeutic zinc supplementation is considered for a long time as a possible option for T cell-mediated autoimmunity [6, 8]. To clarify, whether T cells could be potential targets of zinc supplementation in autoimmune diseases like MS, recently, we investigated the effect of zinc aspartate on T cell activation *in vitro* and on T cell-mediated autoimmunity *in vivo*. We have previously shown that zinc aspartate (Unizink®), a commercially available zinc supplement, is capable of suppressing the proliferation as well as Th1/Th2/Th17 cytokine production of human stimulated T cells and activated mouse splenocytes *in vitro* [10, 11]. Moreover, intraperitoneal (i.p.) administration of a medium-range dose of zinc aspartate in a therapeutic manner led to a significant reduction of the clinical severity of the EAE [10]. Thus, the trace element zinc is the only nontoxic metal which has the special capacity to suppress the proliferation as well as cytokine production of activated T cells and to be highly potent in the active EAE in mice.

Intravenous immunoglobulin (IVIG) preparations contain pooled immunoglobulin G (IgG) from the plasma of approximately thousand blood donors. IVIGs are used in a variety of conditions, especially in replacement therapy of primary and secondary immunodeficiency disorders, in approved autoimmune diseases, and in off-label indications for several autoimmune diseases [12]. IVIGs are currently being used as part of an “off-label” therapy in MS, particularly as a prophylactic approach in pregnant MS patients [13–15]. First studies have shown their efficacy in EAE [16, 17].

Concerning the combined application of IVIG and zinc preparations in EAE, no studies exist as yet. Thus, we wanted to answer the question whether the combination of IVIG and zinc aspartate is effective in EAE as an animal model of MS.

## 2. Materials and Methods

### 2.1. Materials

IVIG (Octagam®) was purchased from Octapharma GmbH (Langenfeld, Germany) and zinc aspartate (Unizink®) from Köhler Pharma GmbH (Alsbach-Hähnlein, Germany). Proteolipid protein (PLP) peptide (p)139–151, corresponding to the mouse sequence (HSLGKWLGHPDKF) was synthesized on a peptide synthesizer and purified by HPLC.

### 2.2. Mice

Female SJL/J mice, age 10–12 weeks, were purchased from JANVIER LABS (Le Genest-Saint-Isle, France) and housed in the animal facilities of the medical faculty of the Otto-von-Guericke-University, Magdeburg. All procedures were conducted according to protocols approved by the Institutional Animal Care and Use Committee (number 42502-2-781 UniMD).

### 2.3. Induction, Treatment, and Evaluation of Active EAE

Female SJL/J mice were immunized subcutaneously (s.c.) in depots distributed over 4 spots across the flanks with 200 *μ*g PLP (p)139–151 in 0.2 ml emulsion consisting of equal volumes of PBS and complete Freund's adjuvant (CFA; Sigma, Taufkirchen, Germany), containing 4 mg/ml of *Mycobacterium tuberculosis* H37Ra (Difco, Detroit, MI). 200 ng pertussis toxin (PTX; List Biological Laboratories, Campbell, CA) was administered intraperitoneally (i.p.) at days 0 and 2 [18].

For therapeutic treatment, the mice received IVIG (10 mg/day), 30 *μ*g/day zinc aspartate or both preparations intraperitoneally (i.p.) from day 11 to day 15 or from day 11 to day 19 after immunization. Equal volumes of phosphate-buffered saline (PBS) served as vehicle controls.

The mice were scored daily for clinical signs of EAE according to the following increasing severity scale: 0: no disease; 1: tail weakness (tail plegia); 2: hindlimb paraparesis and/or weak righting reflex; 3: hindlimb paraplegia; 4: paraplegia with forelimb weakness or paralysis; and 5: moribund animals. Mice with intermediate clinical signs were scored in 0.5 increments. For reasons of animal welfare, the mice were killed when reaching a score of 3 or above. Daily clinical scores were calculated as the average of all individual disease scores of each group [18].

### 2.4. Histological Analysis

For histological analysis, the mice were euthanized at day 20 postimmunization. Spinal cords were removed, fixed in 4% paraformaldehyde, embedded in paraffin, and stained with hematoxylin and eosin (H&E). Thoracic and lumbar spinal cord sections were evaluated, and total numbers of inflammatory foci were determined by an examiner blinded to the treatment status of the animal.

### 2.5. Statistical Analysis

Statistical comparison of EAE disease severity was accomplished by performing a Mann Whitney analysis as described previously [19]. Statistical analyses of the histological data were performed by the ANOVA test using the GraphPad Prism software (version 5.0).

## 3. Results

### 3.1. Influence of Preventive and Therapeutic Application of IVIG on the Clinical Course of EAE

Recently, we could show that i.p. administration of 30 *μ*g/day zinc aspartate in a therapeutic manner led to a significant reduction of the clinical severity of the EAE in SJL/J mice [10].

**Figure 1:**
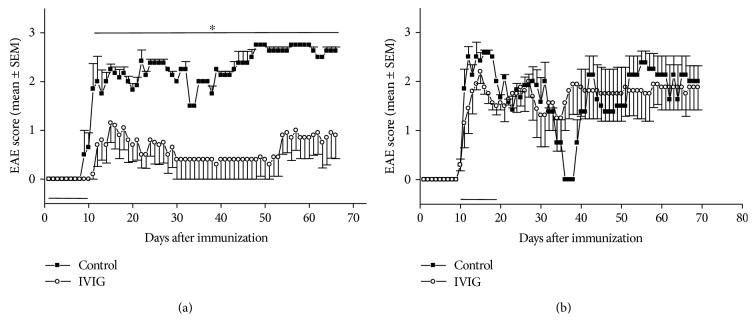
Effects of preventive and therapeutic application of IVIG on clinical signs of EAE. EAE was induced by immunization of SJL/J mice with PLP (p)139–151 as described in Materials and Methods. Mice were treated i.p. in a preventive manner from day 1 to day 10 (a) or in a therapeutic manner from day 11 to day 19 (b) with 10 mg/day IVIG. PBS treatment served as vehicle control. Treatment periods are indicated by the horizontal bar. Clinical disease scores were recorded daily. Data are presented as daily averages ± SEM of disease scores from 5 mice per treatment group. Significance is indicated by the horizontal bar above the curves. ∗ indicates a significance of *p* < 0.05 (Mann Whitney analysis).

In order to characterize the effect of IVIG application in active EAE, SJL/J mice were immunized with PLP (p)139–151. IVIGs (10 mg/day) were given i.p. in a preventive (treated from day 1 to day 10 after immunization) or in a therapeutic manner (treated from day 11 to day 19). PBS treatment served as vehicle control. We observed that the IVIG preparation was capable of diminishing the severity of EAE only in a preventive application (Figure 1(a)). In contrast, therapeutic administration of IVIG alone had no effect on the severity of EAE (Figure 1(b)).

### 3.2. Effects of Therapeutic Application of IVIG, Zinc Aspartate, and the Combination of Both on Clinical Signs of EAE

**Figure 2:**
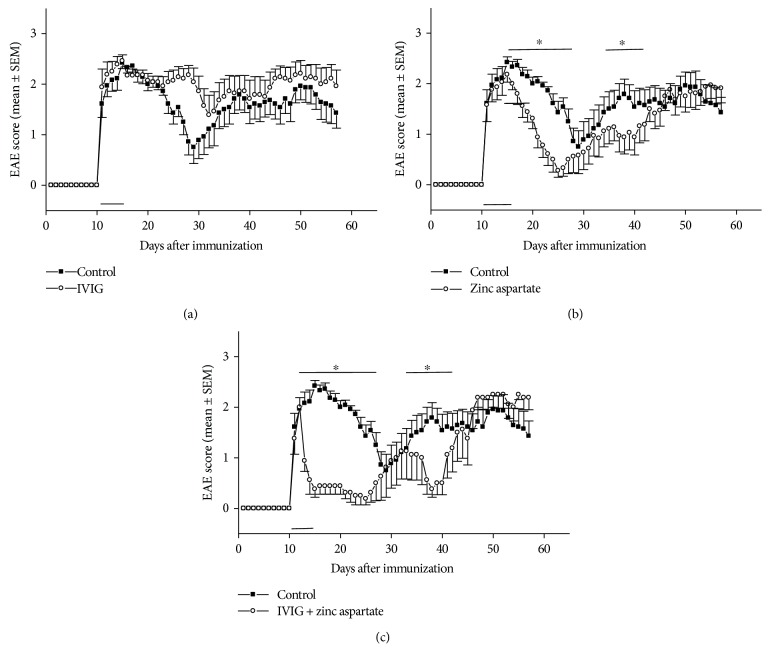
Effects of therapeutic application of IVIG, zinc aspartate, and the combination of both on clinical signs of EAE. EAE was induced by immunization of SJL/J mice with PLP (p)139–151. Mice were treated i.p. from day 11 to day 15 with 10 mg/day IVIG (a), 30 *μ*g/day zinc aspartate (b), or combination of 10 mg/day IVIG and 30 *μ*g/day zinc aspartate (c). PBS treatment served as vehicle control. Treatment periods are indicated by the horizontal bar. Clinical disease scores were recorded daily. Data are presented as daily averages ± SEM of disease scores from 10 mice per treatment group. Significance is indicated as horizontal bar above the curves. ∗ indicates a significance of *p* < 0.05 (Mann Whitney analysis).

Next, we wanted to answer the question whether the therapeutic application of IVIG in combination with zinc aspartate can diminish the severity of the clinical course of EAE. The mice were treated i.p. with 10 mg/day IVIG, 30 *μ*g/day zinc aspartate or with IVIG and zinc aspartate in combination from day 11 to day 15. As shown in Figure 2(c), the combination of IVIG and zinc aspartate caused a rapid remission of the acute phase of EAE and prevented further relapses. The mice treated with IVIG and zinc aspartate showed already in the acute phase of disease a significantly lower severity of the EAE (mean score of 0.5) than the mice of the PBS-treated control group (mean score 2.25). Moreover, the mean EAE score of the treated group was significantly lower than that of the control group in the first relapse of the disease.

As expected, therapeutic IVIG application alone from day 11 to day 15 after immunization showed no positive effect on the severity of the EAE (Figure 2(a)), whereas the therapeutic application of zinc aspartate led to an improvement in the clinical setting in the active phase and first relapse (Figure 2(b)). However, this effect was not as strong as the effect of the combination of IVIG and zinc aspartate.

This observation indicates a synergistic effect of IVIG and zinc in the treatment of relapsing-remitting EAE in mice.

**Figure 3:**
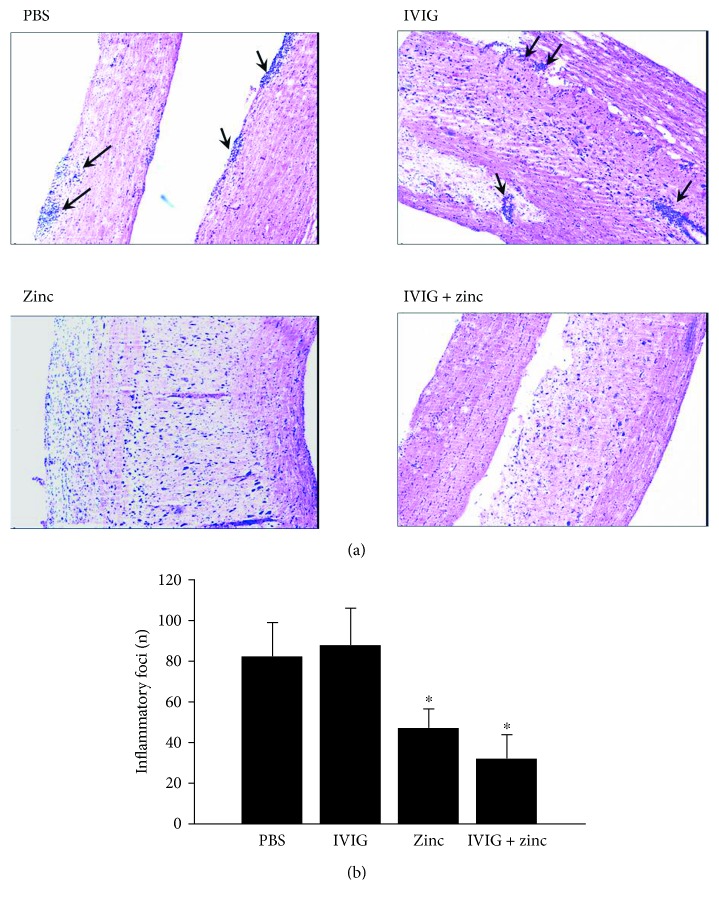
Effect of therapeutic application of IVIG, zinc aspartate, and the combination of both on formation of inflammatory lesions in the CNS of EAE mice. EAE was induced by immunization of SJL/J mice with PLP_139–151_. Mice (*n* = 4 per group) were treated daily i.p. with 10 mg/day IVIG, 30 *μ*g/day zinc aspartate, the combination of both preparations, or vehicle control from day 11 to day 15. Spinal cords were extracted on day 20, fixed in 4% paraformaldehyde, and embedded in paraffin. Sections were stained with H&E. (a) Representative histology of spinal cord longitudinal sections. Inflammatory infiltrations were visualized by H&E staining. The arrowheads show a typical heavy inflammatory cellular infiltration; original magnification ×200. (b) Inflammatory foci in H&E-stained spinal cord cross sections were quantified. Data represent the mean number of inflammatory foci + SEM; ^∗^*p* < 0.05 (ANOVA).

Confirming the clinical data, histopathological analysis of EAE spinal cord tissues showed significantly decreased numbers of total inflammatory foci in EAE mice, which were treated i.p. with zinc aspartate or with the combination of zinc aspartate and IVIG from day 11 to day 15 (Figure 3).

**Figure 4:**
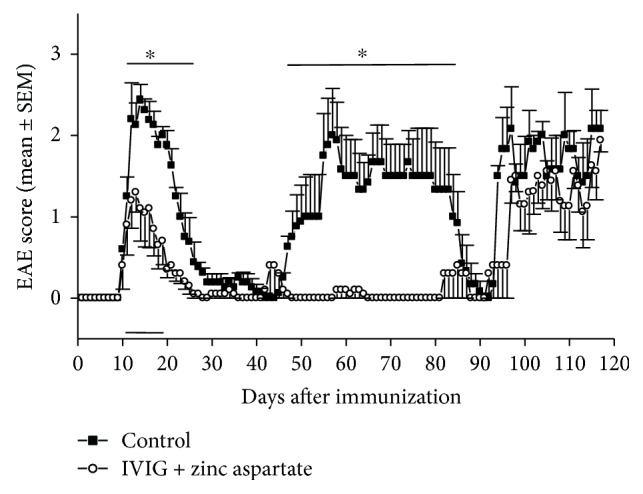
Effects of combined therapeutic application of IVIG and zinc aspartate on clinical signs of EAE. EAE was induced by immunization of SJL/J mice with PLP (p)139–151. Mice were treated i.p. from day 11 to day 19 with 10 mg/day IVIG and 30 *μ*g/day zinc aspartate. PBS treatment served as vehicle control. Treatment periods are indicated by the horizontal bar. Clinical disease scores were recorded daily. Data are presented as daily averages ± SEM of disease scores from 5 mice per treatment group. Significance is indicated by the horizontal bar above the curves. ∗ indicates a significance of *p* < 0.05 (Mann Whitney analysis).

Since therapy over a time period of 5 days already showed a significant improvement of EAE severity, we next investigated the combined effect of IVIG and zinc aspartate in a prolonged treatment protocol from day 11 to day 19 after immunization (Figure 4). In this experiment, the combination showed also a rapid improvement of the clinical picture in the acute phase of EAE (from day 12 to day 27) compared to the PBS control group. In addition, the first relapse of EAE in the group of treated animals was almost completely prevented (from day 47 to day 84). Only after a time period of 100 days after immunization the clinical course of both groups did not show any differences. Overall, these experiments establish the combination of IVIG and zinc as an effective treatment for relapsing-remitting EAE.

## 4. Discussion

In the past twenty years, it has been demonstrated that zinc is a key regulator of the immune system, capable of stimulating or suppressing different immune cells *in vitro* and *in vivo* in a dose- and cell type-dependent manner [6, 8]. Zinc has been reported to affect several cytokine-signalling cascades [20, 21]. We could show that zinc aspartate suppresses proliferation as well as interleukin- (IL-) 2, interferon- (IFN-) *γ*, IL-10, and IL-17 production of stimulated human and mouse T cells *in vitro* [10, 11].

Changes of zinc homeostasis affect both the innate and the adaptive immune system. On one site, impaired immune function due to zinc deficiency can be normalized by therapeutic zinc supplementation [9]. Several authors suggested therapeutic zinc supplementation as a possible option in T cell-mediated autoimmunity [6, 8]. Recently, we could show that i.p. administration of a medium-range dose of 30 *μ*g/day zinc aspartate in a therapeutic manner reduced significantly the clinical severity of the EAE [10]. Moreover, oral administration of 12 *μ*g/day zinc aspartate led to a significant reduction of the clinical severity of the EAE during the relapses of the disease [11].

In the absence of other therapeutic options, IVIG is used as part of an “off-label therapy” in several autoimmune diseases and, e.g., for the postpartum prophylaxis of MS in pregnant women [15, 22]. The work of several groups has well documented that IVIGs modulate the immune system, but the exact mechanism is still not clear. IVIG is shown to block Fc receptors in mononuclear phagocytes, to suppress MHC antigen presentation and antigen recognition by the T cell receptor, to decrease the expression of proinflammatory cytokines, to block autoantibodies, and to induce regulatory T cells [23, 24]. Additionally, a functional role of the immunosuppressive cytokine transforming growth factor-*β* (TGF-*β*) was suggested for IVIG. Kekow et al. [25] reported that substantial amounts of latent TGF-*β* are present in commercially available IVIG preparations.

Comparing the effect of prophylactic and therapeutic IVIG administration on the course of active EAE in SJL/J mice, we found that IVIGs are capable of diminishing the severity of EAE in this animal model only after preventive application. This is in line with studies by other authors [16, 17]. The therapeutic administration of IVIG did not affect the severity of the EAE.

So far, no studies exist concerning the combined application of IVIG and zinc preparations in EAE. Thus, in the present study, we asked the question whether the combination of IVIG and zinc aspartate is effective in active EAE in SJL/J mice. Therefore, we combined the application of IVIG and zinc aspartate in a therapeutic manner and observed a significant reduction of the severity of the disease relapses. The therapeutic treatment with IVIG showed no effect on the acute phase of EAE or the further course. The therapeutic administration of zinc prevented the first relapse of the EAE but had no significant effect on the acute phase of the disease. Interestingly, the combined application of IVIG and zinc caused a rapid remission of the acute phase of EAE and prevented further relapses. Only about 100 days after the therapy, the course of disease of treated mice matched the course of the control group. This therapeutic effect not only was clinically evident from the EAE score but also could be demonstrated histologically. The number of inflammatory foci in the spinal cord was significantly reduced in animals treated with zinc aspartate alone or with the combination of IVIG and zinc compared to the control group. Further studies should be performed to investigate the effect of the combined treatment of zinc aspartate and IVIG after the first and/or second relapse of EAE.

For both MS and EAE, a T cell-mediated pathogenesis, in which Th1 and Th17 cells play a crucial role, is discussed [26, 27]. Both IVIG and zinc preparations are capable of interfering with regulatory proteins of the immune cells. In CD4^+^ T cells, the binding of zinc to STAT3 leads to a change in the *α*-helix structure of this protein, which subsequently prevents STAT3 activation and inhibits differentiation to Th17 cells [28]. IVIGs are shown to suppress Th17 differentiation by inhibiting STAT3 phosphorylation and ROR-*γ*t expression [29–31].

Recently, it was demonstrated in the EAE animal model that IVIG can inhibit the initiation of pathogenic immune response by inhibiting the polarization of naïve T cells into Th17 and Th1 cells, simultaneously increasing regulatory T (Treg) cells [32].

Furthermore, it is known that the immunosuppressive cytokine TGF-*β*1 in the absence of proinflammatory cytokines like IL-6 inhibits the expression of ROR-*γ*t in CD4^+^ T cells and subsequently the differentiation to Th17 cells [33]. The IVIG preparation used in our study contained 11.10 ng/ml latent TGF-*β*1 as measured by a specific ELISA system (data not shown). This is in line with earlier observations [25].

In order to perform its function, latent TGF-*β* must be activated, which is realized *in vivo* especially by various proteases. These include endoproteases like matrix metalloproteases (MMP) [28, 34]. Interestingly, zinc is involved in the regulation of several of these enzymes [35].

The present work shows a significant improvement of the clinical course of EAE under zinc or zinc and IVIG administration. The results suggest that the combination of zinc and IVIG is capable of inhibiting the proliferation of Th1 and Th17 cells and thus suppressing the CNS inflammatory response during EAE. In addition, it was shown that the combination of IVIG and zinc significantly reduces the severity of the EAE in comparison to the administration of zinc aspartate alone.

In addition to Th1 and Th17 cells, CD4^+^CD25^+^FoxP3^+^ Treg cells play also an important role in the pathogenesis of EAE and MS [28, 36, 37]. For this reason, it should be examined in future studies to what extent zinc and IVIG have an effect on the induction of regulatory T cells in the CNS. Moreover, further preclinical and clinical studies will help to clarify whether the combined application of IVIG and zinc can be beneficial for the treatment of T cell-mediated autoimmune diseases like MS.

## Data Availability

The data used to support the findings of this study are included within the article.

## Abbreviations

CFA: Complete Freund's adjuvant
CNS: Central nervous system
EAE: Experimental autoimmune encephalomyelitis
H&E: Hematoxylin and eosin
HPLC: High-performance liquid chromatography
IL: Interleukin
IFN: Interferon
IgG: Immunoglobulin G
i.p.: Intraperitoneal
IVIGs: Intravenous immunoglobulins
MS: Multiple sclerosis
PBS: Phosphate-buffered saline
PBMC: Peripheral blood mononuclear cells
PLP: Proteolipid protein
PTX: Pertussis toxin
TGF-*β*: Transforming growth factor-*β*
Treg: Regulatory T cells.
